# Comparison of professional and everyday wearable technology at different body positions in terms of recording gait perturbations

**DOI:** 10.1371/journal.pdig.0000553

**Published:** 2024-08-30

**Authors:** Lea Feld, Lena Schell-Majoor, Sandra Hellmers, Jessica Koschate, Andreas Hein, Tania Zieschang, Birger Kollmeier

**Affiliations:** 1 Department of Medical Physics and Acoustics, Medical Physics and Cluster of Excellence Hearing4all, Carl von Ossietzky University, Oldenburg, Germany; 2 Department for Health Services Research, Assistance Systems and Medical Device Technology, Carl von Ossietzky University, Oldenburg, Germany; 3 Department for Health Services Research, Geriatric Medicine, Carl von Ossietzky University, Oldenburg, Germany; The University of Hong Kong, HONG KONG

## Abstract

Falls are a significant health problem in older people, so preventing them is essential. Since falls are often a consequence of improper reaction to gait disturbances, such as slips and trips, their detection is gaining attention in research. However there are no studies to date that investigated perturbation detection, using everyday wearable devices like hearing aids or smartphones at different body positions. Sixty-six study participants were perturbed on a split-belt treadmill while recording data with hearing aids, smartphones, and professional inertial measurement units (IMUs) at various positions (left/right ear, jacket pocket, shoulder bag, pants pocket, left/right foot, left/right wrist, lumbar, sternum). The data were visually inspected and median maximum cross-correlations were calculated for whole trials and different perturbation conditions. The results show that the hearing aids and IMUs perform equally in measuring acceleration data (correlation coefficient of 0.93 for the left hearing aid and 0.99 for the right hearing aid), which emphasizes the potential of utilizing sensors in hearing aids for head acceleration measurements. Additionally, the data implicate that measurement with a single hearing aid is sufficient and a second hearing aid provides no added value. Furthermore, the acceleration patterns were similar for the ear position, the jacket pocket position, and the lumbar (correlation coefficient of about 0.8) or sternal position (correlation coefficient of about 0.9). The correlations were found to be more or less independent of the type of perturbation. Data obtained from everyday wearable devices appears to represent the movements of the human body during perturbations similar to that of professional devices. The results suggest that IMUs in hearing aids and smartphones, placed at the trunk, could be well suited for an automatic detection of gait perturbations.

## Author summary

More and more researchers are tackling the issue of falls in older people, a significant problem leading to hospitalization and loss of independence. Falls often result from improper responses to the loss of balance. Our aim is to identify unsteady walking using everyday devices like smartphones and hearing aids, which have sensors to record movement patterns. In our study, participants were tested with these devices placed in various positions on their bodies while walking on a special treadmill, which can create unbalanced situations. Our findings suggest that hearing aids and smartphones, particularly when located on the trunk, could be able to detect and prevent falls by monitoring walking patterns.

## Introduction

Falls in older adults are not only a leading cause of injuries and hospitalization [1], but also often lead to immobilization and a worsening of the affected person’s quality of life [2, 3]. Hence, falls are a significant health problem [4, 5]. Over the last few years, more and more research in fall detection has been done and increasingly implemented using body-worn inertial measurement units (IMUs). The most frequently used measurement positions in current research are at the waist and the trunk, as shown in the review by Schwickert et al. [6]. However, there are also studies that deal with fall detection where the sensor was attached to less common measurement positions, e.g. the head [7–9]. Since researchers have shown that accelerations measured at the head reliably show regular patterns related to gait [10], there have also been studies using an ear-worn sensor to detect gait characteristics, activities, and pathological gait patterns [8, 11, 12]. This could be used to make gait analysis accessible to a broad population at risk through the use of hearing aids, like in [8], and to better depict everyday life situations. Previous studies have shown that smartphones placed in different locations, such as in the pants pocket, on the waist or in the handbag, can be used for gait analyses and fall detection [13–20].

More desirable than the detection of a fall, however, is the prevention of the fall event itself to avoid the negative consequences. Early detection of an increased risk of falling can increase the success of preventive measures and can preserve a person’s quality of life [21]. Among other things (e.g. environment, behavior, biology etc.) near-falls are related to the risk of falling, since falls are often a consequence of improper reactions to perturbations whereby, in a near-fall situation, the compensating movement was still sufficient to prevent the fall. The detection of these, potentially preceded by gait perturbations like slips, trips, or missteps is gaining attention in research [22–26]. Moreover, perturbation training is increasingly investigated and used in people with Parkinson’s disease and stroke to improve gait and reduce their risk of falling [27–31]. Alike for fall detection IMUs are often used for the detection of gait perturbations. As shown in the overview by Pang et al. [32] the IMUs were placed at the chest and/or waist position, which are referred to as the standard positions. In a study on construction workers Zhang et al. [33] demonstrated the feasibility of smartphones and artificial neural networks to measure near-miss falls. To our knowledge, there are no studies to date that have investigated near-falls like slips, trips, and missteps, using everyday wearable devices in high-risk older adults. Such research could contribute to identify gait irregularities at an early stage and estimate the fall risk.

This work investigates the suitability of IMUs in hearing aids and smartphones at different positions for potentially detecting gait perturbations in comparison to professional IMUs. The investigation of hearing aids is particularly important in terms of their potential for early detection of increased risk of falls as well as fall events through the integration of IMUs. They have the advantage of a clearly defined attachment position and are an everyday device, especially for older people with age-related hearing loss who also have an increased risk of falling.Currently, to our best knowledge, three hearing aid manufacturers already offer hearing aids with included motion sensors: Starkey [8], Phonak [34] and Signia [35]. An initial step towards achieving gait perturbation detection with everyday technology is to ensure that the IMUs in smartphones and hearing aids measure acceleration data similar to that of professional IMUs. With regard to smartphones, there are already studies comparing acceleration data measured with smartphones to data from IMUs [36] at the same position (lumbar). However, to our knowledge, there are no studies to date that compare the acceleration data recorded with hearing aids to data from professional equipment. Visual inspection and cross-correlation were used to compare everyday wearable measurement devices (hearing aids and smartphones) and their attachment positions (right/left ear, shoulder bag, jacket pocket, pants pocket) to professional IMUs attached to standard positions (lumbar and sternum). Analyses were also conducted individually for each perturbation to identify any differences in suitability based on the type of perturbation.

## Materials and methods

This study was approved by the medical ethics committee of the University of Oldenburg (number 2021-093). All participants provided written informed consent to voluntarily participate in the study and received financial compensation on an hourly basis.

### Participants

For this exploratory study, the aim was to recruit a heterogeneous group of adults. A total of 66 adults (46 females and 20 males, mean age: 57.8 years [18–87 years], mean BMI: 25.8 kg/m² [18.4–36.2 kg/m²], mean gait speed: 1.07 m/s [0.42–1.67 m/s], SPPB (Short Physical Performance Battery) mean score: 10.76 [7–12], TUG (Timed Up and Go) mean score: 2.8 [1–3]) above the age of 18 years participated in the study. Participants with various conditions were included, for example, six people with herniated discs, four people with spinal stenosis, one person with a lumbar vertebral fracture and two people with dizziness, while others had no gait-affecting conditions. Persons were excluded, who had acute diseases requiring special medical observation during the experiment, who were not able to sign the informed consent themselves, or could not walk without walking aids for at least 20 min. Due to technical limitations, persons with a body height larger than 2 m, or with a body weight above 135 kg, or a waist circumference of more than 120 cm were excluded from participation.

### Experimental setup

#### Experimental protocol

**Fig 1:**
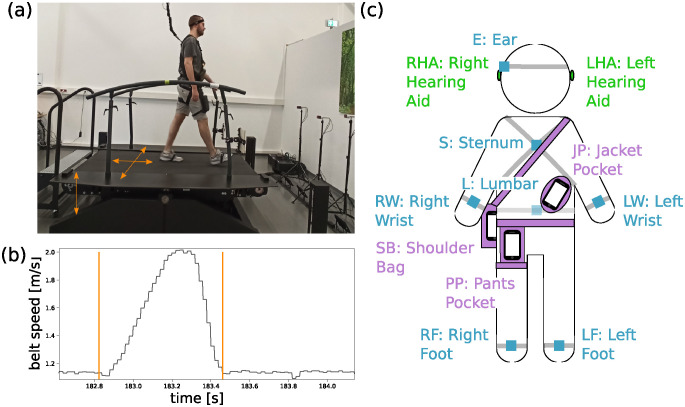
Perturbation treadmill and measurement devices. (a) The perturbation treadmill is used to induce various perturbations by moving the treadmill in different directions. The different directions (medial-lateral, anterior-posterior, tilt) are indicated by orange arrows. (b) This figure shows the right belt speed of the treadmill over time during an exemplary “slip right” disturbance. (c) The participants were equipped with everyday wearable (green and purple) and professional (blue) devices at different positions.

The measurement took place in a lab environment on a perturbation treadmill (MGait Motek Medical B.V., Amsterdam, the Netherlands) with a built-in split-belt. It offers the possibility to control the left and right belt separately. The treadmill belt could be accelerated and decelerated, the treadmill platform could be moved in medial-lateral direction and could be tilted, as shown in Fig 1a by the orange arrows.

Using these features, nine different gait disturbances were applied in a standardized way: slips and trips of the left and right leg, sway to the left, and right as well as positive and negative pitch. For the “slip right/left” and the “trip right/left” perturbation (anterior-posterior direction), the treadmill belt was accelerated to 180% or decelerated to 40% of the individual walking speed, respectively, for about 0.42 s with 3 m/s², while the walking speed was kept constant for the other belt. These disturbances can be associated with, e.g., slipping on a wet floor or tripping by getting caught with a foot on a branch while walking in the forest. An example for the treadmill belt acceleration (slip) is given in Fig 1b. For the “trip both” perturbation both treadmill belts were decelerated with 9 m/s² to 0 m/s for 0.12 s, which can be associated with, e.g., an emergency stop in public transportation. The “sway right/left” disturbance in medial-lateral direction was introduced by a displacement of the treadmill by 5 cm to the right/left side with 3 m/s². This can be associated with, e.g., standing sideways in a bus that accelerates or decelerates. The “pitch positive/negative” disturbance was generated by tilting the treadmill by ± 5° for 1 s and can be associated with, e.g., encountering an overlooked ramp or curb.

Prior to the perturbation trials, participants selected their preferred walking speed during overground walking. Subsequently, the treadmill speed was set to 50% of their chosen walking speed and gradually incremented until participants once again reached their preferred walking speed. After familiarizing themselves with the treadmill for about one minute, each participant underwent three perturbation trials involving nine different perturbations presented in a pseudo-random order. In each trial, the perturbations were triggered by the initial foot contact, determined via the built-in force plates, of the dominant leg. The time interval between successive perturbations varied between 20 and 30 seconds. To ensure the safety of the participants, they wore a safety harness during the recordings to avoid injuries in case of an actual fall event on the treadmill. Participants had a two-minute break between the trials.

#### Measurement devices

While walking on the treadmill, participants’ movements were captured using both everyday wearable and professional devices, as illustrated in Fig 1c. As everyday wearable technology, smartphones (Samsung Galaxy A52; sampling frequency *f*_SP_ = 100 Hz) and hearing aids (Starkey Livio AI 2400; sampling frequency *f*_HA_ = 104±4 Hz) were used. The accelerometers in these devices measured the acceleration of the respective body part to which the device was attached. To cover typical wearing positions for smartphones (pants pocket, jacket pocket, shoulder bag), three identical smartphones were placed in a bag on the upper right thigh, the left side of the trunk, and in a cross-shoulder bag positioned on the left shoulder, crossing over to the right side (see Fig 1c). To avoid overloading the participants and restricting their freedom of movement, each bag was attached only to one side, yet positioned to ensure coverage of both halves of the body in total. To emphasize more realistic scenarios, the smartphones were not securely fixed in their respective pockets. Data was recorded using an app developed for this purpose. Hearing aids were positioned on both ears and connected via Bluetooth to an iPhone SE, which recorded the transferred data using an app from the hearing aid manufacturer. It’s important to note that the hearing aids were used solely as measurement devices and did not provide any amplification or other hearing support. In addition, professional inertial measurement units (IMUs) were used to compare the measured data. Six IMU devices (Opal V1, Mobility Lab™ (ML), APDM, Inc., Portland, OR, USA; sampling frequency *f*_APDM_ = 128 Hz) were placed at commonly used positions for gait analysis (right/left foot, right/left wrist, lumbar, sternum). Additionally, one IMU device was placed at the right ear with a headband to provide a reference signal for the right hearing aid. Signals from the IMUs were recorded using the provided software (Mobility Lab from the company APDM).

### Data processing

Only acceleration data was analyzed, as initial results indicated its greater promise in addressing the research question compared to the gyroscope data, although the latter remains a potential starting point for further investigations. The unfiltered raw data were re-sampled to 300 Hz, which is the frequency of the force plates integrated into the treadmill. This data was then used to calculate the absolute acceleration *a* for each device according to Eq (1), where ${\vec{a}}_{x}$, ${\vec{a}}_{y}$, and ${\vec{a}}_{z}$ represents the acceleration in the three spatial directions, respectively.

$$\begin{array}{c} a=\sqrt{{\vec{a}}_{x}^{2}+{\vec{a}}_{y}^{2}+{\vec{a}}_{z}^{2}} \end{array}$$ (1)

#### Synchronization

To compare data from all different devices, synchronization of the devices was necessary. The treadmill provides a signal at its start, which, with the help of a USB-controlled relay phidget and the external synchronization option, ensures the synchronization of the IMU (Opal) devices with the treadmill. The smartphones were synchronized by a specific event, in this case by a jump that the study team performed before the start of the test. All smartphones and three IMU devices were placed in a box, and three jumps were performed while holding this box with both arms outstretched. The measured absolute accelerations of the three IMU devices were averaged and used as a reference to temporally synchronize the smartphone data using cross-correlation.

The hearing aid data did not provide an actual timestamp. For this reason, synchronization was performed for each trial individually and not via a jump event by correlating the data with the data recorded by the IMU at the ear position. To account for the different sampling frequencies of the devices, the hearing aid frequency was slightly varied simultaneously to maximize the correlation between the recorded events. For the two hearing aid pairs used, the following median sampling frequencies were found: $f_{\text{HA},l}^{1}$ = 100.320 ± 0.010 Hz, $f_{\text{HA},r}^{1}$ = 107.828 ± 0.010 Hz for pair one, and $f_{\text{HA},l}^{2}$ = 102.793 ± 0.010 Hz, $f_{\text{HA},r}^{2}$ = 100.3625 ± 0.0050 Hz for pair two.

#### Definition of perturbation excerpts

The start time of each perturbation was provided by the treadmill. The end of each perturbation was determined manually by identifying the timestamp when the changed parameter (e.g., belt speed) returned to the pre-perturbation mean plus-minus the standard deviation. In Fig 1b, the perturbation range (orange lines) as well as the change of the belt speed is shown for an exemplary “slip right” perturbation.

For the subsequent analysis of individual perturbations, data from the start of the perturbation to the manually identified end of the perturbation plus three times the mean step time of each participant was included. The step time of each participant was calculated as the average of the time duration between successive initial contacts, recorded by the force plates integrated into the treadmill. The calculation of the mean step time did not differentiate between left and right steps. Here the paper by Debelle et al. [37] was referred to, which found that participants need about five steps after a treadmill-generated slip perturbation to take at least one step back within the normal margin of stability at heel strike. So by including the perturbation and also only three steps afterward, the direct reaction of the participant to the perturbation was captured.

#### Median maximum cross-correlation

As a quantitative measure to compare the different devices and positions, the median maximum cross-correlation values for the absolute acceleration data were used. For this the full cross-correlation was calculated after (*r*_*i*,*j*_)^*k*^(*τ*) = [(*a*_i_*a_*j*_)(*τ*)]^*k*^ for all trials *k* of all participants for each combination of two sensor positions *i*, *j* ∈ (RF, LF, L, S, RW, LW, E, RHA, LHA, SB, JP, PP), see Fig 1c for abbreviations. Subsequently the maximum of each full cross-correlation with respect to *τ* was determined and defined as the maximum cross-correlation value ${\left(r_{i,j}^{\text{max}}\right)}^{k}$ for the data of the respective sensor positions *i* and *j* for trial number *k*. This results in one correlation matrix for each trial of each participant. From this, one median correlation matrix was calculated after Eq (2) by taking the median over all participants and trials for each matrix cell, respectively. For the calculation of the median matrix only trials that are complete for all devices, i.e., without any dropouts or missing data, were included, which lead to *k* = (1, …, 143) trials from 58 different participants.

$$\begin{array}{c} \left({\tilde{r}}_{i,j}\right)={\text{med}}_{k}\{{\left(r_{i,j}^{\text{max}}\right)}^{k}\}={\text{med}}_{k}\{{\text{max}}_{\tau }({\left[\left(a_{i}*a_{j}\right)\left(\tau \right)\right]}^{k})\} \end{array}$$ (2)

## Results

### Visual inspection

**Fig 2:**
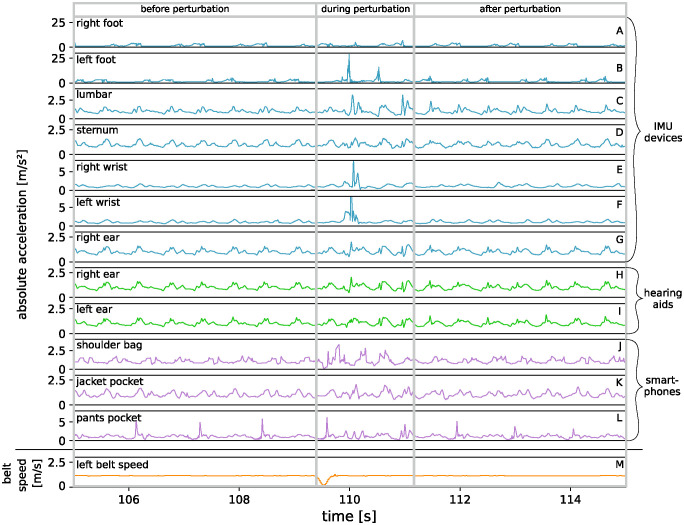
Visual inspection of exemplary absolute acceleration data. The figure shows absolute accelerations for professional inertial measurement units (IMUs), hearing aids and smartphones at different body positions (see Fig 1c for detail) exemplary for one participant for the “trip both” perturbation. The lowest panel shows the belt speed of the left treadmill belt.

First, the data was visually inspected. Fig 2 illustrates an example of the measured absolute acceleration in m/s² (see Eq (1)) before, during, and after a “trip both” perturbation during one trial for one participant. The data is presented for all measurement devices at different body positions, showing the dependence on time in seconds. The first seven panels (A-G) show the measured data of the IMU devices (indicated in Fig 2 by blue lines) placed at the right foot, left foot, lumbar, sternum, right wrist, left wrist and right ear. In the two panels below (H, I), the data of the hearing aids positioned at the right and left ear are displayed (green lines), and below that (J-L), the data of the smartphones (purple lines) placed in a shoulder bag, jacket pocket and pants pocket. The lowest panel (M) of the figure shows the measured belt speed in m/s of the left treadmill belt. The belt speed indicates the point in time when the perturbation was introduced, i.e., the speed of both treadmill belts was reduced to 0 m/s for “trip both”. The three temporal areas, “before perturbation“, “during perturbation” and “after perturbation“, were defined as follows. The area “before perturbation” shows an excerpt of normal walking on the treadmill until the start of the perturbation. The area “during perturbation” covers the range of the perturbation plus three additional steps of the participant, as explained in Sec Data processing. The area “after perturbation” follows directly after that and contains several seconds of walking without perturbation. First of all, it is noticeable that the measured acceleration data of the right hearing aid (H) seems to be nearly identical to the data recorded with the IMU at the same position (G). Furthermore, the data of the left hearing aid (I) is also very similar to the data recorded with the IMU at the right ear (G). Therefore, these three device positions will be combined in the term “ear position” for the following description.

In the first part of the data, i.e., “before perturbation”, a regular pattern is evident in each device. This periodic pattern corresponds to and is characteristic for normal walking of a person on a treadmill. However, the patterns differ in their periodicity, distinctness and in the pattern itself. The devices at the left and right foot show, as expected, alternating data where each peak indicates a step with the left or right foot, respectively. The data recorded at the lumbar, sternum, ear (which also includes the hearing aids), wrists, shoulder bag and jacket pocket positions show a periodic pattern, where each period corresponds to one step, regardless of whether it was a left or a right step. The periodic pattern therefore appears with twice the frequency of the foot positions. The data recorded at the pants pocket position show three peaks in this first area (“before perturbation”), each corresponding to a peak in the data for the right foot. This can be explained by the fact that the pants pocket was attached to the right thigh.

At the beginning of the second area, the “trip both” perturbation was introduced, as shown in the lower panel (M) of Fig 2, depicting the belt speed of the treadmill. In this area, i.e., “during perturbation”, the periodic pattern of the data is interrupted for most devices and positions, and irregular sequences occur. The shape and characteristics of the irregularities differ between sensor positions. The data of the device attached to the left foot shows two prominent peaks, contrasting with the device attached to the right foot. This can be explained by the fact that the perturbation was triggered on the initial contact of the right foot, which was the participants dominant leg. Hence, the first pronounced peak corresponds to the quick compensating step that the person took with the left foot to maintain balance. Additionally, the devices at the left and right wrist recorded high peaks, depicting that the participant snatched up the arms as a reaction to the perturbation. Furthermore, higher accelerations at the lumbar and shoulder bag position and three smaller peaks at the pants pocket position can be seen. Whereas the perturbation does not have such a pronounced effect on the accelerations measured at the sternum, ear, and jacket pocket position.

In the third area, i.e., “after perturbation”, the acceleration data converge back to the baseline pattern of the first area. The person regains their balance and has recovers from the introduced perturbation.

### Cross-correlation

As a quantitative measure to compare the different devices and positions, the median maximum cross-correlation matrix ${\tilde{r}}_{i,j}$ for the absolute acceleration data, calculated according to Eq (2) for all sensor positions *i* and *j*, was used. Since each correlation matrix is symmetrical with respect to the matrix diagonal, only the upper or lower triangular matrix is displayed for each condition. All correlations were found to be highly significant with p-values smaller than 0.01.

#### Whole trials

**Fig 3:**
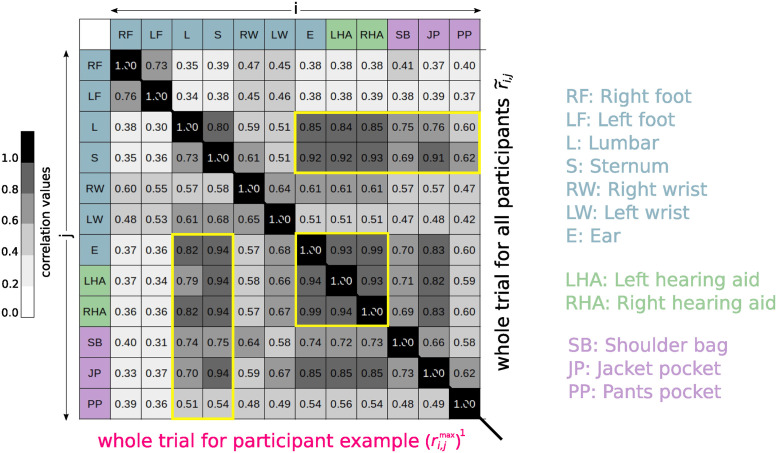
Cross-correlation coefficients for whole trials. Median maximum cross-correlation coefficients ${\tilde{r}}_{i,j}$ between all used devices for all whole trials (upper triangular matrix) and the maximum cross-correlation coefficients ${\left(r_{i,j}^{\text{max}}\right)}^{1}$ for the whole trial from which the excerpt in Fig 2 was taken (lower triangular matrix) were calculated. The yellow boxes indicate the comparison of the attachment positions of the everyday wearable devices in relation to the standard measurement positions as well as the hearing aids to the professional IMU at the ear position.

In Fig 3, the calculated cross-correlation matrices for whole perturbation trials, which include normal walking as well as nine perturbations for each trial, are displayed. The upper triangular matrix shows the median matrix over all trials ${\tilde{r}}_{i,j}$, while the lower triangular matrix shows the maximum correlation matrix ${\left(r_{i,j}^{\text{max}}\right)}^{1}$ over the trial including the exemplary excerpt in Fig 2. For the sake of simplicity, *k* = 1 was chosen for this specific trial. The correlation coefficients were interpreted after [38] as very strong (0.9–1), strong (0.7–0.89), moderate (0.4–0.69), weak (0.1–0.39) and negligible (0.0–0.1) correlation. The first thing that can be noted is that there is at least a moderate correlation between the absolute acceleration data of everyday wearable devices and every other device and position, except for the IMUs at the feet, where only a weak correlation was found.

The data of the right hearing aid (everyday wearable technology) and the IMU (professional technology) placed at the same position yields a very strong correlation with a value of ${\tilde{r}}_{E,\text{RHA}}=0.99$. This value is very close to one which means that the data have a high positive linear relationship. A very strong correlation can also be observed for the data of the left hearing aid, where the correlation to the professional IMU at the right ear yields a value of ${\tilde{r}}_{E,\text{LHA}}=0.93$. Given the substantial similarity in the data for the right and left hearing aid and the IMU at the right ear, these positions will be merged (“ear position”) in terms of the correlation coefficient below. The following comparison focuses only on the attachment positions of the everyday wearable devices, namely the ear, shoulder bag, jacket pocket, and pants pocket position, in relation to standard measurement positions, i.e., lumbar and sternum. This restricts the sensor positions *i* and *j* to *i* ∈ (E, JP, SB, PP) and *j* ∈ (L, S) for the following description.

The data recorded at the ear position, encompassing both hearing aids and the IMU at the right ear, shows a strong correlation of around ${\tilde{r}}_{E,L}\approx 0.84$ with the lumbar position and a very strong correlation of around ${\tilde{r}}_{E,S}\approx 0.92$ with the sternum position. Similar results are observed for the acceleration data measured at the jacket pocket position, with a correlation value of ${\tilde{r}}_{\text{JP},L}$ = 0.76 and ${\tilde{r}}_{\text{JP},S}=0.91$ with respect to the lumbar and sternum position, respectively. The shoulder bag, despite being loosely attached to the body, exhibits a quite similar correlation coefficient with the lumbar position (${\tilde{r}}_{\text{SB},L}=0.75$) as the jacket pocket. However, the shoulder bag shows a lower correlation with the sternum position (${\tilde{r}}_{\text{SB},S}=0.69$) compared to the jacket pocket. For the pants pocket position, the lowest correlation coefficients to the standard positions with regard to the everyday wearable technology were found. The data measured at the pants pocket position yield just moderate correlation to the standard positions lumbar and sternum with values of around ${\tilde{r}}_{\text{PP},L}\approx {\tilde{r}}_{\text{PP},S}\approx 0.60$.

The maximum cross-correlation values for the exemplary participant, depicted in the lower triangular matrix of Fig 3 are similar to the median values (upper triangular matrix), which means that the same correlation pattern generally emerges. Nevertheless, a few differences can be noted. Deviations greater than or equal to 0.05 were found for lumbar position to the new mobile positions with correlation values of ${\left(r_{\text{LHA},L}^{\text{max}}\right)}^{1}=0.79$, ${\left(r_{\text{JP},L}^{\text{max}}\right)}^{1}=0.70$ and ${\left(r_{\text{PP},L}^{\text{max}}\right)}^{1}=0.51$. In comparison to that correlation values of ${\tilde{r}}_{\text{LHA},L}=0.84$, ${\tilde{r}}_{\text{JP},L}=0.76$ and ${\tilde{r}}_{\text{PP},L}=0.60$ were found for the median over all participants. For the sternum position deviations greater than or equal to 0.05 were found for ${\left(r_{\text{SB},S}^{\text{max}}\right)}^{1}=0.75$ and ${\left(r_{\text{PP},S}^{\text{max}}\right)}^{1}=0.54$ in comparison to ${\tilde{r}}_{\text{SB},S}=0.69$ and ${\tilde{r}}_{\text{PP},S}=0.62$.

#### Perturbation conditions

To investigate if the correlations change by including only data recorded “during perturbation”, the cross-correlation for each perturbation was calculated, and the difference to the median maximum cross-correlation matrix for the whole trial was determined as

$$\begin{array}{c} {\left(\Delta {\tilde{r}}_{i,j}\right)}_{\text{perturbation}}={\left({\tilde{r}}_{i,j}\right)}_{\text{perturbation}}-{\left({\tilde{r}}_{i,j}\right)}_{\text{wholetrial}}. \end{array}$$ (3)

**Fig 4:**
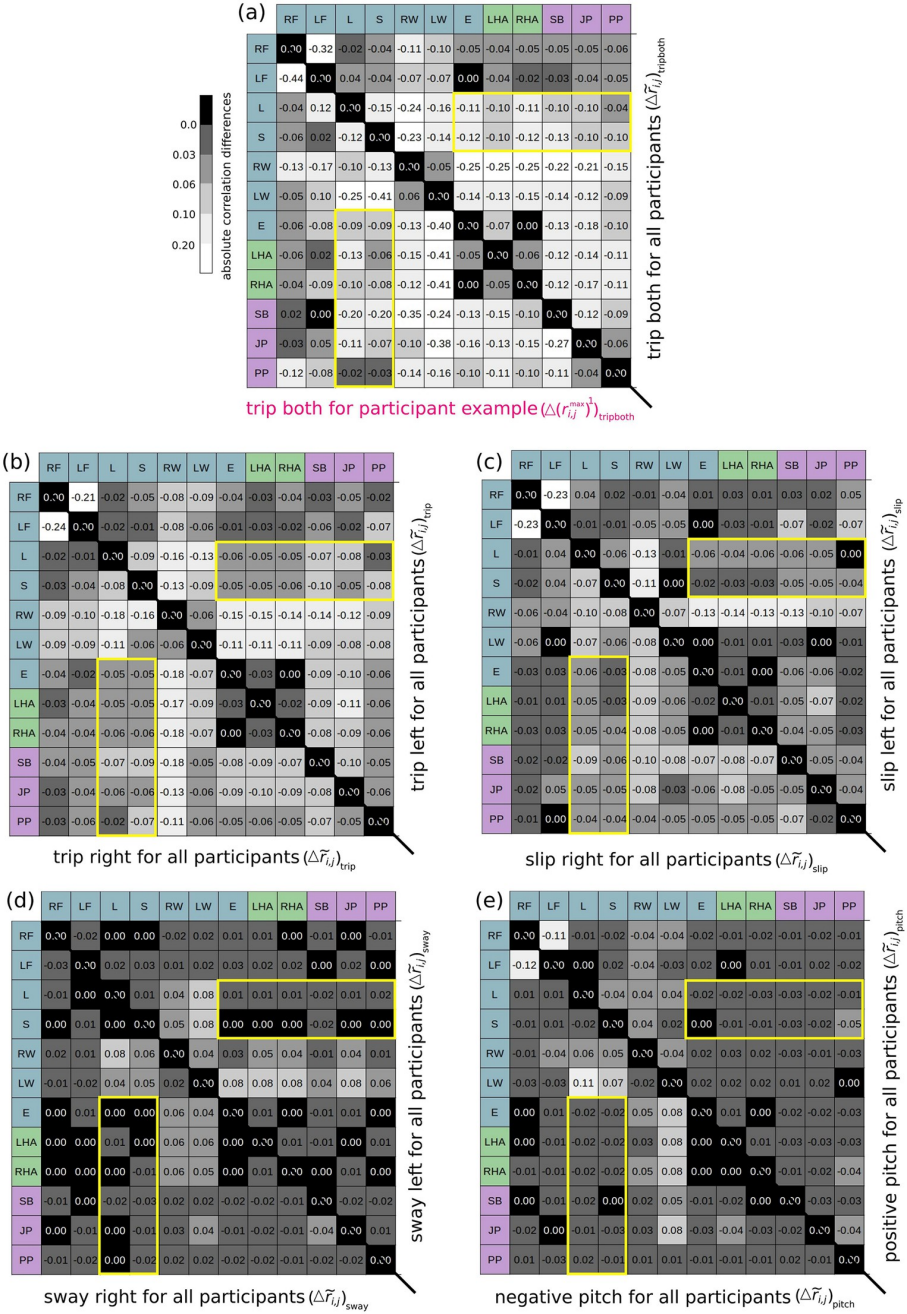
Difference in the median maximum cross-correlation matrices for different perturbation conditions. Difference in the median maximum cross-correlation coefficients for all whole trials and each perturbation condition $\Delta {\tilde{r}}_{i,j}$, respectively. The yellow boxes indicate the comparison of the attachment positions of the everyday wearable devices in relation to the standard measurement positions. (a) upper triangular matrix: “trip both”; lower triangular matrix: the “trip both” perturbation shown in the excerpt in Fig 2. (b) upper triangular matrix: “trip left”; lower triangular matrix: “trip right”. (c) upper triangular matrix: “slip left”; lower triangular matrix: “slip right”. (d) upper triangular matrix: “sway left”; lower triangular matrix: “sway right”. (e) upper triangular matrix: “positive pitch”; lower triangular matrix: “negative pitch”.

The resulting matrices for the differences are shown in Fig 4a–4e. Even if the correlation values were calculated for all sensor positions, the focus here is only on the sensor positions *i* ∈ (E, JP, SB, PP) and *j* ∈ (L, S) (indicated by the yellow boxes). It first should be noticed, that the difference in the median maximum cross-correlation matrices for different conditions, is in general quite small, with all absolute difference values being less than 0.13. Moreover nearly all difference values are negative, which means that the correlation values are lower for the perturbation conditions than for the whole trial.

Although the difference values for all perturbations are therefore close to each other, different patterns can be recognized for the different perturbations. The difference between the median maximum correlation values for the “pitch” and “sway” conditions to the whole trial, see Fig 4d and 4e, yields with $-0.05\leq {\left(\Delta {\tilde{r}}_{i,j}\right)}_{\text{pitch}}\approx {\left(\Delta {\tilde{r}}_{i,j}\right)}_{\text{sway}}\leq 0.03$ the smallest values, which means that the matrix values are very similar.

The matrices for the “trip” and “slip” conditions (see Fig 4b and 4c) show difference values that are a little bit higher with differences of $-0.1\leq {\left(\Delta {\tilde{r}}_{i,j}\right)}_{\text{trip}}\approx {\left(\Delta {\tilde{r}}_{i,j}\right)}_{\text{slip}}\leq 0$. The “trip both” condition, shown in the upper diagonal matrix in Fig 4a, exhibits the greatest difference regarding the correlation values compared to the whole trial condition with up to ${\left(\Delta {\tilde{r}}_{i,j}\right)}_{\text{tripboth}}$ = –0.13. Note, that the lower triangular matrix of Fig 4a, which shows the maximum cross-correlation difference for one exemplary participant for the “trip both” condition, i.e. the second area depicted in Fig 2, yields similar results. However small deviations over 0.05 were found for the shoulder bag to the lumbar and sternum ${\left(\Delta {\left(r_{\text{SB},L}^{\text{max}}\right)}^{1}\right)}_{\text{tripboth}}={\left(\Delta {\left(r_{\text{SB},S}^{\text{max}}\right)}^{1}\right)}_{\text{tripboth}}=-0.2$ and for the pants pocket and the sternum position ${\left(\Delta {\left(r_{\text{PP},S}^{\text{max}}\right)}^{1}\right)}_{\text{tripboth}}=-0.03$, where the median values yield ${\left(\Delta {\tilde{r}}_{SB,L}\right)}_{\text{tripboth}}=-0.1$, ${\left(\Delta {\tilde{r}}_{SB,S}\right)}_{\text{tripboth}}=-0.13$ and ${\left(\Delta {\tilde{r}}_{PP,S}\right)}_{\text{tripboth}}=-0.1$.

## Discussion

The visual inspection of the absolute acceleration in Fig 2 showed how the acceleration data differ for normal walking on the treadmill and for the compensatory reaction of the participant as a response to an external perturbation. Additionally, the median maximum cross-correlation values were calculated for the whole trials of all participants as well as all perturbation conditions to compare everyday wearable measurement devices (hearing aids and smartphones) and their attachment positions (right/left ear, shoulder bag, jacket pocket, pants pocket) to professional IMUs placed at standard positions (lumbar and sternum).

### Hearing aids vs. IMU at ear position

Visual inspection of the acceleration data recorded with the right hearing aid and the IMU device on the right ear shows a striking similarity of the data. This observation is supported by the very strong correlation (0.99) found between these two positions for the whole trial, indicating almost identical data convergence. As both devices were positioned almost at the same place, one would theoretically expect the data to match exactly if both devices had the same capabilities to measure acceleration. However, minor deviations are due to slight mounting variations and sensor specifications. From this it can be concluded that the devices are indeed equally good at measuring acceleration data, which emphasizes the potential of utilizing sensors in hearing aids for head acceleration measurements.

In addition, a very strong correlation (0.93) was found between the left hearing aid and the IMU on the right ear or the right hearing aid. Although both hearing aids are on opposite sides of the body, the head is a relatively rigid unit, which means that the movement of the left ear mirrors that of the right ear almost exactly. Consequently, one would expect very similar acceleration data for both positions, which was also observed. This implies that both left and right hearing aids capture nearly identical information, suggesting that the choice of which ear to measure movement on may not significantly impact the results. This would also mean that a measurement with two hearing aids would not provide any real gain in information compared to a measurement with only one hearing aid, so that a measurement with a single hearing aid could already be sufficient.

### Ear position vs. standard positions

Between the absolute acceleration recorded at the ear position and those recorded at the standard positions, the sternum and lumbar, a strong to very strong correlation was identified. This means that the movement of the ear and therefore the head is very similar to the movement of the lumbar and the sternum. In particular, the signals of the sternum and the ear showed a very strong correlation. To support the results, a look was taken at the principal component analysis (PCA) of the time series data. To apply a PCA to time series data, each observation is treated as a separate variable and arranged in a matrix. The PCA is performed on this matrix to obtain the principal components, which represent the main trends and patterns in the data over time. In this case the PCA was performed on the absolute accelerations measured at the different body positions for each trial and participant (see data in S1 and S2 Dataset). When considering the first principal components the ear position (IMU at the right ear as well as left and right hearing aid) only has a major contribution to the first principal component for most participants. Hereby the first component already explains about 50% of the variance in the data. In fact the lumbar and sternal position also contribute to the first principal component to a similar extent as the ear position. The component could therefore be interpreted as a representation of the movement of the torso. The common contributions to this component also suggest that these positions similarly depict the gait movement during the whole trial on the treadmill. These findings align well with the results reported in [39], where it was asserted that accelerations measured at the head during normal walking represent a somewhat dampened version of the accelerations observed in the lower torso. This observation is also consistent with earlier studies ([40–42]). The phenomenon can be explained by the constant effort of the human body to maintain a stable state. Hereby the upper body’s damping capacity ensures a continuous minimization of head oscillations while walking. Consequently, this results in acceleration patterns for the ear position that are similar but slightly damped compared to those at the lumbar or sternum.

### Common smartphone positions vs. standard positions

Besides the ear position, also positions where a smartphone is usually worn were examined: the jacket pocket, the shoulder bag, and the pants pocket position. Upon analyzing the visual data, a consistent pattern was observed between the data captured at the jacket pocket position and that recorded at the sternum and lumbar positions. Notably, the data from the jacket pocket position exhibits a greater similarity to the sternum position than to the lumbar position. This pattern is also reflected in the correlation coefficients, where a very strong correlation with the sternum position and a strong correlation with the lumbar position were found for the jacket pocket. One possible explanation for the higher correlation with the sternum compared to the lumbar position might be the jacket pocket’s frontal placement on the trunk, while the lumbar sensor is at the back. Conversely, this could also explain why the shoulder bag, located at the back, has a strong correlation with the lumbar and only a moderate correlation with the sternum. The data for the smartphone in the pants pocket yields only moderate correlation for both the lumbar and sternum position.

To support the results a look at the principal component analysis was also taken here. The jacket pocket and the pants pocket position contribute to the first principal component in approximately equal measure as the lumbar and sternal position, whereby the pants pocket position has a slightly lower contribution. This joint contribution to a principal component could explain the high correlation values between the positions. If the first component is again assumed as the movement of the torso, it also makes sense that the pants pocket makes a smaller contribution than the other positions, as it is attached to the thigh and not directly to the torso. So the data for the pants pocket position is less influenced by the torso movement and more influenced by the foot movement than the other smartphone positions. However, the correlations for the shoulder bag cannot be so easily explained by the principal components, as there is no really consistent pattern across the different participants. It should be noted that the correlations calculated for the shoulder bag with the lumbar and sternum show the greatest variance, twice as great as for the other mobile positions. This can be explained by the fact that the shoulder bag could be fitted in the least standardized way. An attempt was made to place the upper end of the bag on the subject’s hip bone by eye, but the movement of the bag depended very much on the subject’s stature, but also on the gait movement. For some people, the bag was at the back all the time, whereas for others it slipped to the side when walking. Hence, the measured data differ much more across the participants.

The results indicate that a smartphone especially in a jacket pocket seems to be able to detect perturbations with a similar precision and using the same algorithms as professional IMUs worn at the most-used sensor positions on the trunk.

### Whole trials vs. perturbation conditions

The median maximum cross-correlation coefficients were calculated not only for the whole trials but also for all perturbation conditions and compared with the results for the whole trials. It was found that the correlation coefficients between the new and standard sensor positions for all perturbation conditions did not deviate very much from those for the whole trial, as can be seen from the small difference values in Fig 4. This means that the relationship between the different body positions does not really seem to depend on the type of perturbation. However, it can be observed that almost all differences are negative, i.e. the absolute correlation values are lower than for the whole trial. Although the data for whole trials also contain the perturbations, undisturbed walking between the perturbations accounts for the majority of the data. During undisturbed walking, the body moves in a very stable, regulated sequence in which the movement propagates through the body in a certain way. If the body is then disturbed by an external unexpected perturbation, faster compensatory reactions occur. For these, the propagation of the movement through the body is expected to be different from normal walking.

Although it was stated that the deviations are so small that it can be said that the perturbations do not have a major influence on the correlation values, systematic differences can be found among the perturbations. The greatest difference was found for the “trip both” condition and the smallest differences were found for the “pitch” and “sway” conditions. This can be explained by the different nature of the perturbations due to speed and direction. The “trip both” perturbation is the most extreme one due to the short time scale during which both belts are completely stopped. This perturbation can be associated with a full stop, which means that the participant must perform a strong compensatory response to maintain his balance. In such a reaction to an unexpected event that throws one off balance, the highest deviation from the normal gait is expected. The “pitch” and “sway” perturbation affect both sides of the body equally (“pitch”) or did not differ much between the sides of the body (“sway”). In most cases, people found the perturbation less challenging and were able to regain their balance with just a few non-extreme movements. This means that the movements are not so different from those during normal walking, which is reflected in the small difference of the median maximum cross-correlation coefficients. The perturbations that were applied just on one side at a time, namely the “trip left/right” and “slip left/right” perturbation led to greater reactions, and with that higher differences, than for the “pitch” or “sway” but were not as extreme as for the “trip both” condition.

### Individual vs. median results

The visual inspection in Fig 2 is only a short excerpt for the “trip both” perturbation of a trial from an exemplary participant. In order to find out whether the results of this one participant are representative and thus transferable to the median for all participants, the maximum cross-correlation was also calculated for one whole trial of this participant and the “during perturbation” section shown in Fig 2. The data for this one specific trial and the median data for all trials as well as for the “trip both” perturbation (see the lower triangular matrix in Figs 3 and 4a, respectively) showed in general similar correlation patterns regarding the correlation values. It can be concluded from this that the data in Fig 2 seems to reflect the average behavior of all participants, allowing well-founded conclusions to be drawn from the observations. The deviations can be explained by the fact that each person has an individual gait pattern, which can lead to variations in the correlation values between the different positions. In addition, the participants also react to the perturbations with different strategies, so that deviations can also be expected for the “trip both” condition, as observed here.

## Conclusion

In conclusion, the study shows strong correlations between data measured with hearing aids, smartphones placed at the trunk and standard sensor positions (lumbar and sternum) during gait and different perturbation conditions. The results provide promising insights into the potential use of ear-level sensors, such as hearing aids, for gait analysis and link mobile health data to the established literature on professional gait analysis. The data presented indicates promising device positions, such as the ears and a jacket pocket, that could be used for the detection of possible indicators of gait perturbations, as the data is similar to standard positions and thus similar algorithms could be used. It is important to note that a weak correlation to the standard positions at lumbar height and sternum does not preclude the automatic detection of gait perturbations with this device and at this specific position. Although these results are encouraging, further research is needed to validate long-term data, especially in older individuals with conventional fall reporting. In addition, further research is needed on the objective identification of gait perturbations with the help of everyday technology using algorithms. This could also help to validate the recorded signals from everyday technology with respect to real events of gait instability as the current study only considered laboratory conditions and no specific algorithm for gait instability detection. Overall, this opens new pathways for research and practical application of everyday wearable devices in healthcare.

## Supporting information

S1 Dataset Principal components for the acceleration data of the whole trials of all participant. The first eight principal components for the absolute acceleration data were calculated for the whole trial of all participants.
(PKL)

S2 Dataset Variance explained by the principal components for the acceleration data of the whole trials for all participant. Variance explained by the first eight principal components for the absolute acceleration data were calculated for the whole trial of all participants.
(PKL)

S3 Dataset Maximum cross-correlation values for whole trials. Maximum cross-correlation values for 58 whole trials from 143 participants.
(PICKLE)

S4 Dataset Maximum cross-correlation values for negative pitch. Maximum cross-correlation values for the negative pitch perturbations from 58 whole trials from 143 participants.
(PICKLE)

S5 Dataset Maximum cross-correlation values for positive pitch. Maximum cross-correlation values for the positive pitch perturbations from 58 whole trials from 143 participants.
(PICKLE)

S6 Dataset Maximum cross-correlation values for slip left. Maximum cross-correlation values for the slip left perturbations from 58 whole trials from 143 participants.
(PICKLE)

S7 Dataset Maximum cross-correlation values for slip right. Maximum cross-correlation values for the slip right perturbations from 58 whole trials from 143 participants.
(PICKLE)

S8 Dataset Maximum cross-correlation values for sway left. Maximum cross-correlation values for the sway left perturbations from 58 whole trials from 143 participants.
(PICKLE)

S9 Dataset Maximum cross-correlation values for sway right. Maximum cross-correlation values for the sway right perturbations from 58 whole trials from 143 participants.
(PICKLE)

S10 Dataset Maximum cross-correlation values for trip both. Maximum cross-correlation values for the trip both perturbations from 58 whole trials from 143 participants.
(PICKLE)

S11 Dataset Maximum cross-correlation values for trip left. Maximum cross-correlation values for the trip left perturbations from 58 whole trials from 143 participants.
(PICKLE)

S12 Dataset Maximum cross-correlation values for trip right. Maximum cross-correlation values for the trip right perturbations from 58 whole trials from 143 participants.
(PICKLE)

## References

[1] Shankar KN, Liu SW, Ganz DA. Trends and characteristics of emergency department visits for fall-related injuries in older adults, 2003–2010. Western J Emerg Med. 2017;18(5):785. doi: 10.5811/westjem.2017.5.33615 28874929 PMC5576613

[2] Moreland J, Richardson J, Chan DH, O’Neill J, Bellissimo A, Grum RM, Shanks L. Evidence-based guidelines for the secondary prevention of falls in older adults. Gerontology. 2003;49(2):93–116. doi: 10.1159/000067948 12574670

[3] Ambrose AF, Paul G, Hausdorff JM. Risk factors for falls among older adults: a review of the literature. Mauritas. 2013;75(1):51–61. 23523272 10.1016/j.maturitas.2013.02.009

[4] Tinetti ME. Preventing falls in elderly persons. New England journal of medicine. 2003;348(1):42–49. 12510042 10.1056/NEJMcp020719

[5] Organization WHO. Falls. 2021. Available from: https://www.who.int/news-room/fact-sheets/detail/falls.

[6] Schwickert L, Becker C, Lindemann U, Maréchal C, Bourke A, Chiari L, et al. Fall detection with body-worn sensors: a systematic review. Zeitschrift für Gerontologie und Geriatrie. 2013;46(8). 24271251 10.1007/s00391-013-0559-8

[7] Lindemann U, Hock A, Stuber M, Keck W, Becker C. Evaluation of a fall detector based on accelerometers: A pilot study. Medical and Biological engineering and computing. 2005;43:548–551. doi: 10.1007/BF02351026 16411625

[8] Rahme M, Folkeard P, Scollie S. Evaluating the accuracy of step tracking and fall detection in the Starkey Livio artificial intelligence hearing aids: A pilot study. American journal of audiology. 2021;30(1):182–189. doi: 10.1044/2020_AJA-20-00105 33284647

[9] Guilbeault-Sauvé A, De Kelper B, Voix J. Man down situation detection using an in-ear inertial platform. Sensors. 2021;21(5):1730. doi: 10.3390/s21051730 33802287 PMC7959136

[10] Cromwell R, Wellmon R. Sagittal plane head stabilization during level walking and ambulation on stairs. Physiotherapy Research International. 2001;6(3):179. doi: 10.1002/pri.226 11725599

[11] Diao Y, Ma Y, Xu D, Chen W, Wang Y. A novel gait parameter estimation method for healthy adults and postoperative patients with an ear-worn sensor. Physiological measurement. 2020;41(5):05NT01. doi: 10.1088/1361-6579/ab87b5 32268319

[12] Burgos CP, Gärtner L, Ballester MAG, Noailly J, Stöcker F, Schönfelder M, et al. In-ear accelerometer-based sensor for gait classification. IEEE Sensors Journal. 2020;20(21):12895–12902. doi: 10.1109/JSEN.2020.3002589

[13] Habib MA, Mohktar MS, Kamaruzzaman SB, Lim KS, Pin TM, Ibrahim F. Smartphone-based solutions for fall detection and prevention: challenges and open issues. Sensors. 2014;14(4):7181–7208. doi: 10.3390/s140407181 24759116 PMC4029687

[14] Brajdic A, Harle R. Walk detection and step counting on unconstrained smartphones. Proceedings of the 2013 ACM international joint conference on Pervasive and ubiquitous computing; 2013. p. 225–234.

[15] Mrozek D, Koczur A, Małysiak-Mrozek B. Fall detection in older adults with mobile IoT devices and machine learning in the cloud and on the edge. Information Sciences. 2020;537:132–147. doi: 10.1016/j.ins.2020.05.070

[16] Leu FY, Ko CY, Lin YC, Susanto H, Yu HC. Fall detection and motion classification by using decision tree on mobile phone. Smart Sensors Networks. Elsevier; 2017. p. 205–237.

[17] Fang SH, Liang YC, Chiu KM. Developing a mobile phone-based fall detection system on android platform. 2012 Computing, Communications and Applications Conference. IEEE; 2012. p. 143–146.

[18] Dai J, Bai X, Yang Z, Shen Z, Xuan D. PerFallD: A pervasive fall detection system using mobile phones. 2010 8th IEEE International Conference on Pervasive Computing and Communications Workshops (PERCOM Workshops). IEEE; 2010. p. 292–297.

[19] Harari Y, Shawen N, Mummidisetty CK, Albert MV, Kording KP, Jayaraman A. A smartphone-based online system for fall detection with alert notifications and contextual information of real-life falls. Journal of neuroengineering and rehabilitation. 2021;18(1):124. doi: 10.1186/s12984-021-00918-z 34376199 PMC8353784

[20] Tsinganos P, Skodras A. A smartphone-based fall detection system for the elderly. Proceedings of the 10th International Symposium on Image and Signal Processing and Analysis. IEEE; 2017. p. 53–58.

[21] Mellone S, Tacconi C, Schwickert L, Klenk J, Becker C, Chiari L. Smartphone-based solutions for fall detection and prevention: the FARSEEING approach. Zeitschrift fur Gerontologie und Geriatrie. 2012;45(8):722. doi: 10.1007/s00391-012-0404-5 23184298

[22] Trkov M, Chen K, Yi J, Liu T. Inertial sensor-based slip detection in human walking. IEEE Transactions on Automation Science and Engineering. 2019;16(3):1399–1411. doi: 10.1109/TASE.2018.2884723

[23] Wang S, Miranda F, Wang Y, Rasheed R, Bhatt T. Near-fall detection in unexpected slips during over-ground locomotion with body-worn sensors among older adults. Sensors. 2022;22(9):3334. doi: 10.3390/s22093334 35591025 PMC9102890

[24] Karel JM, Senden R, Janssen JE, Savelberg H, Grimm B, Heyligers I, et al. Towards unobtrusive in vivo monitoring of patients prone to falling. 2010 Annual International Conference of the IEEE Engineering in Medicine and Biology. IEEE; 2010. p. 5018–5021. 10.1109/IEMBS.2010.5626232 21096022

[25] Weiss A, Shimkin I, Giladi N, Hausdorff JM. Automated detection of near falls: algorithm development and preliminary results. BMC research notes. 2010;3:1–8. doi: 10.1186/1756-0500-3-62 20205708 PMC2845599

[26] Handelzalts S, Alexander NB, Mastruserio N, Nyquist LV, Strasburg DM, Ojeda LV. Detection of real-world trips in at-fall risk community dwelling older adults using wearable sensors. Frontiers in medicine. 2020;7:514. doi: 10.3389/fmed.2020.00514 32984385 PMC7492551

[27] Klamroth S, Steib S, Gaßner H, Goßler J, Winkler J, Eskofier B, et al. Immediate effects of perturbation treadmill training on gait and postural control in patients with Parkinson’s disease. Gait & Posture. 2016;50:102–108. doi: 10.1016/j.gaitpost.2016.08.020 27591395

[28] Coelho DB, de Oliveira CEN, Guimarães MVC, Ribeiro de Souza C, dos Santos ML, de Lima-Pardini AC. A systematic review on the effectiveness of perturbation-based balance training in postural control and gait in Parkinson’s disease. Physiotherapy. 2022;116:58–71. doi: 10.1016/j.physio.2022.02.005 35550488

[29] Mansfield A, Schinkel-Ivy A, Danells CJ, Aqui A, Aryan R, Biasin L, et al. Does Perturbation Training Prevent Falls after Discharge from Stroke Rehabilitation? A Prospective Cohort Study with Historical Control. Journal of Stroke and Cerebrovascular Diseases. 2017;26(10):2174–2180. doi: 10.1016/j.jstrokecerebrovasdis.2017.04.041 28579506 PMC5600819

[30] Alayat MSM, Almatrafi NA, El Fiky AAR, Elsodany AM, Shousha TM, Basuodan R. The effectiveness of perturbation-based training in the treatment of patients with stroke: a systematic review and meta-analysis. Neuroscience Insights. 2022;17:26331055221114818. doi: 10.1177/26331055221114818 35910084 PMC9329815

[31] Handelzalts S, Kenner-Furman M, Gray G, Soroker N, Shani G, Melzer I. Effects of perturbation-based balance training in subacute persons with stroke: a randomized controlled trial. Neurorehabilitation and neural repair. 2019;33(3):213–224. doi: 10.1177/1545968319829453 30767613

[32] Pang I, Okubo Y, Sturnieks D, Lord SR, Brodie MA. Detection of near falls using wearable devices: a systematic review. Journal of geriatric physical therapy. 2019;42(1):48–56. doi: 10.1519/JPT.0000000000000181 29384813

[33] Zhang M, Cao T, Zhao X. Using smartphones to detect and identify construction workers’ near-miss falls based on ANN. Journal of construction engineering and management. 2019;145(1):04018120. doi: 10.1061/(ASCE)CO.1943-7862.0001582

[34] Solveig, Christina Voss. Hearing on the go! n.d. Available from: https://audiologyblog.phonakpro.com/hearing-on-the-go/.

[35] WS Audiology A/S. Help keep your customers performing at their best. 2024. Available from: https://www.signia-pro.com/en/connectivity/my-wellbeing/.

[36] Nishiguchi S, Yamada M, Nagai K, Mori S, Kajiwara Y, Sonoda T, et al. Reliability and validity of gait analysis by android-based smartphone. Telemedicine and e-Health. 2012;18(4):292–296. doi: 10.1089/tmj.2011.0132 22400972

[37] Debelle H, Harkness-Armstrong C, Hadwin K, Maganaris CN, O’Brien TD. Recovery from a forward falling slip: measurement of dynamic stability and strength requirements using a split-belt instrumented treadmill. Frontiers in sports and active living. 2020;2:82. doi: 10.3389/fspor.2020.00082 33345073 PMC7739594

[38] Schober P, Boer C, Schwarte LA. Correlation coefficients: appropriate use and interpretation. Anesthesia & Analgesia. 2018;126(5):1763–1768. doi: 10.1213/ANE.0000000000002864 29481436

[39] Kavanagh J, Morrison S, Barrett R. Coordination of head and trunk accelerations during walking. European journal of applied physiology. 2005;94:468–475. doi: 10.1007/s00421-005-1328-1 15827734

[40] Menz HB, Lord SR, Fitzpatrick RC. Acceleration patterns of the head and pelvis when walking on level and irregular surfaces. Gait & Posture. 2003;18(1):35–46. doi: 10.1016/S0966-6362(02)00159-5 12855299

[41] Winter DA. Biomechanics and motor control of human gait: normal, elderly and pathological. 1991

[42] Winter DA, Ruder GK, MacKinnon CD. Control of balance of upper body during gait. In: Multiple muscle systems: biomechanics and movement organization. Springer; 1990. p. 534–541.

